# Enhanced spectroelectrochemistry with lossy-mode resonance optical fiber sensor

**DOI:** 10.1038/s41598-023-42853-0

**Published:** 2023-09-19

**Authors:** Monika Janik, Katarzyna Lechowicz, Emil Pituła, Jakub Warszewski, Marcin Koba, Mateusz Śmietana

**Affiliations:** 1 Institute of Microelectronics and Optoelectronics, Warsaw University of Technology, Koszykowa 75, 00-662 Warsaw, Poland; 2 National Institute of Telecommunications, Szachowa 1, 02-894 Warsaw, Poland

**Keywords:** Fibre optics and optical communications, Optical sensors

## Abstract

Spectroelectrochemical (SEC) measurements play a crucial role in analytical chemistry, utilizing transparent or semitransparent electrodes for optical analysis of electrochemical (EC) processes. The EC readout provides information about the electrode's state, while changes in the transmitted optical spectrum help identify the products of EC reactions. To enhance SEC measurements, this study proposes the addition of optical monitoring of the electrode. The setup involves using a polymer-clad silica multimode fiber core coated with indium tin oxide (ITO), which serves as both the electrode and an optical fiber sensor. The ITO film is specifically tailored to exhibit the lossy-mode resonance (LMR) phenomenon, allowing for simultaneous optical monitoring alongside EC readouts. The LMR response depends on the properties of the ITO and the surrounding medium's optical properties. As a result, the setup offers three types of interrogation readouts: EC measurements, optical spectrum analysis corresponding to the volume of the analyte (similar to standard SEC), and LMR spectrum analysis reflecting the state of the sensor/electrode surface. In each interrogation path, cyclic voltammetry (CV) experiments were conducted individually with two oxidation–reduction reaction (redox) probes: potassium ferricyanide and methylene blue. Subsequently, simultaneous measurements were performed during chronoamperometry (CA) with the sensor, and the cross-correlation between the readouts was examined. Overall, this study presents a novel and enhanced SEC measurement approach that incorporates optical monitoring of the electrode. It provides a comprehensive understanding of EC processes and enables greater insights into the characteristics of the analyte.

## Introduction

With advancements in various fields such as medical diagnostics, and food or pharmaceutical analysis, novel analytical methods are expected to offer enhanced functionalities and information about the properties of the analyte^1–5^. Often, the methods make the detection of chemical compounds or products of their reactions possible. At best, to make the analysis cost-effective, from the instrumentation point of view, the methods should rely on equipment already available in laboratories. Among the most common analytical methods are electrochemical (EC) and optical, where electric charge transfer and emitted radiant energy or absorption are measured, respectively^3^. To gather enhanced data sets, these two measurements, i.e., optical and EC, are conducted simultaneously creating an approach called spectroelectrochemistry (SEC)^6–10^. In contrast to separate optical and EC analysis, besides the cross-correlation of the obtained information, SEC collects real-time information on the reactants, in situ electrogenerated intermediates, and/or reaction products involved in electron transfer processes^11^. Therefore, SEC is often used for studies on new materials, especially nanomaterials^9^, formation and identification of chemical or biological complexes^12^, as well as reactions and processes characterization^13^.

In a SEC setup a standard EC monitoring of an electric charge transfer at the electrode surface is combined with a spectroscopic cell capable of optical measurements of an analyte in a proximity of the electrode. The EC measurements are generally performed in a three-electrode configuration consisting of a working electrode (WE), at which (mostly) products of reactions are being created, a counter electrode (CE), and a reference electrode (RE). The optical path in SEC is typically aligned perpendicularly to the surface of the WE and may differ in length and beam size dependent on the type of the spectrophotometric cell used and optical elements such as e.g., collimating lens. To make the WE optically transparent or semitransparent, platinum or gold thin wires and meshes, or glass slides coated with EC-active transparent conductive oxides (TCOs), such as indium tin oxide (ITO), are often used. TCOs offer lower than their metallic counterparts attenuation of light passing through the analyte and electrode’s surface^10,14^.

Over the past decades various approaches for the light-analyte interactions in a volume were proposed^6,15^. Among them different waveguide structures were investigated, including those based on optical fibers, where guided light was used for detecting changes in the optical path^16,17^. Optical fibers offer many advantages such as compact size, flexibility in their positioning within the sensing system and easiness in coupling with light source and detector, as well as immunity to electromagnetic interference, and low cost. Moreover, a fused silica optical fiber shows easiness of its surface modification, making it eligible for novel sensing applications. One of the approaches based on the optical fiber for SEC has been reported in^16^. The optical fiber sensor was placed alongside the light path and submerged in the analyte. The optical detection in this case was based on the attenuation changes resulting from total internal reflection. The fiber core section, where the cladding had been removed, was surrounded by a gold mesh serving as the WE. Thus, the fiber structure in the proposed setup was only supporting the gathering of optical properties of the analyte surrounding the fiber. Given the size of the sensing elements (8 cm long exposed fiber core covered by 10 × 100 mm gold mesh), it is worth mentioning that the measurement cell required a relatively large volumes of the analyte. Such a solution, with no additional surface modifications of exposed core has severely limited sensitivity to changes of surrounding analyte and may not be applicable when only small volumes are available. Furthermore, the placement of the gold mesh may differ from sensor to sensor, which may influence the repeatability of the measurements. In later studies, the sensor was based on 400 µm core multimode optical fiber with 7.5-cm-long core section nanocoated with 80 nm ITO layer^18^. In this case, the fiber structure was utilized for both EC measurements and detection of changes in optical properties of the analyte measured as a transmission of the fiber. Besides the length of the sensor that limits its application to large volumes, it must be noted that application of an EC potential to ITO alters concentration charge of a layer near its surface and affects observed optical effect itself^19^. This effect, on top of changes in optical properties of the analyte, should be considered when analyzing performance of the ITO-based opto-EC devices.

In this work we propose an enhanced SEC setup where a dual-domain (optical and EC) optical-fiber-based sensor is directly used as the WE while optical properties of the analyte are measured separately as in SEC. The sensor was obtained in reflective (probe-like) configuration, where only short core section is coated with ITO and immersed in the analyte. The properties of ITO nanocoating are tuned to satisfy the conditions for obtaining lossy-mode resonance (LMR) in the desired range of the reflected spectrum^20^. Among other thin films already used for getting LMR^21–28^, ITO film offers a set of unique properties such as low electrical resistivity, high optical transparency, high EC activity, and durability proven for heavy experimental usage^29,30^. Furthermore, due to an EC-activity of the ITO, a single ITO-LMR structure merges optical and EC domains (EC-LMR). It allows acquisition of extra information on the state of the WE surface and therefore, additional cross-verification of the SEC results. The ITO-LMR sensors have already been reported for monitoring of EC processes^31^, including electrodeposition^32,33^, as well as label-free biosensing^34,35^. However, as the LMR depends on properties of the nanocoating, i.e., its thickness and optical properties, but also properties of the analyte surrounding the nanocoating its application in the SEC setup may be highly beneficial^36^. The LMR-based and spectrophotometric measurements are performed in separate optical paths. This creates a new form of two perpendicularly oriented spectral channels with the EC activation (S^2^EC). Performance of the system has been verified by Cyclic Voltammetry (CV) and Chronoamperometry (CA) in electrolytes containing redox systems such as potassium ferricyanide or methylene blue. The obtained results were compared with those for standard SEC configurations where platinum mesh and ITO-coated glass slide were applied as an electrode with no capability for optical monitoring of the WE.

## Experimental details

### Fabrication of the ITO-LMR sensor

**Figure 1:**
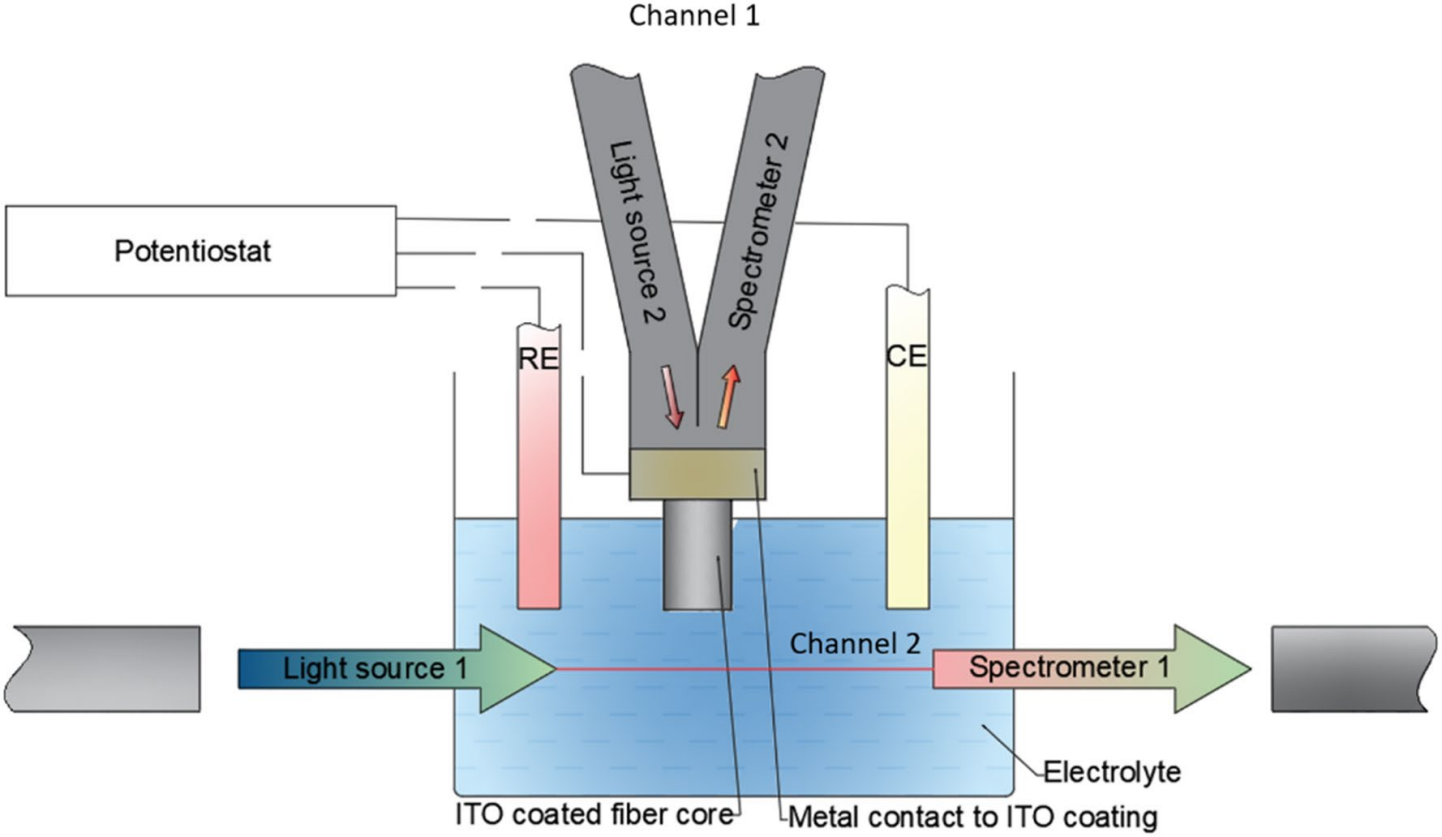
A schematic representation of a S^2^EC (LMR sensor a WE). The elements are not to scale.

Used in this work ITO-LMR sensor was based on a polymer-clad silica (PCS, φ_core_ = 380 μm) multimode optical fiber. First, a set of 20 cm long fibers was prepared. After cleaving the fibers with SETI-ATI multimode fiber cleaver, both end-faces were flat polished with a thin grading polishing disks (9, 3 and 0.3 μm) using Radian (Krelltech) polishing setup. Next, the fiber was cleaned in isopropanol, installed in a sample holder, and prepared for further deposition of thin films. Since the sensors were designed for reflective (probe-like) configuration to efficiently guide light reflected at one of the fiber end-faces, an aluminum film was deposited on one of the fiber ends using DC magnetron sputtering technique^28^. Next, from the side of an aluminum-coated end-face, the polymer cladding was removed with a fiber stripper, exposing there a 2.5-cm-long section of the core, which side surface was cleaned with isopropanol. Further, the core was coated with ITO in a magnetron sputtering process which used a 2-inch ITO target (In2O3:SnO2—90:10 wt%) and was powered by Bdiscom BDS-HF-200 AFP RF source (13.56 MHz, 150 W). The coating buildup procedure included a half turn rotation of a fiber along its long axis to obtain uniform nanocoating. The deposition conditions, including process time and pressure, have a strong impact on the ITO properties. During preliminary EC study, length of the exposed core section and deposition pressure, were altered between 15–35 mm and 0.3–0.5 Pa, respectively. Schematic representation of the LMR sensor in the S^2^EC setup is shown in Fig. 1.

### SEC and S^2^EC setups

A schematic of the experimental setup used for measurements in this work is shown in Fig. 1. Different types of WE can be accommodated in the setup due to application of a standard quartz cuvette (12.5 × 12.5 × 35 mm external dimensions with inner dimensions of 5 × 10 mm). Cuvette is placed in a holder (Ocean Optics) designed for spectrophotometric measurements (optical channel 1, Ch1). The holder is supported by two collimating lenses placed between the cuvette and FT400EMT optical fiber patch cords (Thorlabs) leading the light in and out of the holder. A light beam with a diameter of 3 mm is transmitted through cuvette parallel to its shorter edge (5-mm-long optical path) and 10 mm above its bottom. The transmission via the cuvette was monitored in the wavelength range of 200–1000 nm using DH-2000-BAL deuterium-halogen lamp (Ocean Optics) and an optical fiber USB4000 spectrometer (Ocean Optics). WEs applied in standard SEC, such as ITO-coated glass and platinum mesh, were placed in the light beam, but the LMR electrode (optical channel 2, Ch2), due to its small size, could be placed in various positions vs the beam. In this work ITO-LMR WE was positioned just above the light beam. As counter (CE) and reference electrode (RE), a Pt wire and a Ag/AgCl (3 M KCl) have been used, respectively, and placed in the cuvette above the beam. The EC setup was controlled by EmStat3+ potentiostat (Palmsens) supported by PSTrace software. Two oxidation–reduction reaction (redox) probes were used for verification of the system performance, namely a K3[Fe(CN)6] (FC) diluted in 0.1 M KCl solution and a methylene blue (MB) diluted in 0.1 PBS. Scan rate was set to 50 mV/s.

LMR measurements in the wavelength range of 375–1040 nm were performed using HL-2000 tungsten light source (Ocean Optics) and an optical fiber USB4000 spectrometer (Ocean Optics). Interrogation of the LMR in Ch2 was performed with BFY400 bifurcated fiber supported by a BFT1 bare fiber bullet-type terminator (both from Thorlabs). Simultaneous interrogation and data acquisition in all three channels (optical Ch1, Ch2 and EC) was controlled by an in-house developed MATLAB script. It must be noted that the optical interrogation of the sensor/electrode (optical measurements in Ch2) are possible only when the LMR sensor is applied as the WE, but not when a Pt mesh or an ITO-coated glass slide are used. For the sake of clarity, the enhanced SEC supported by the LMR is noted as S^2^EC, while standard approach with other WEs is called as SEC.

## Results and discussion

### Identification of SEC measurement conditions

**Figure 2:**
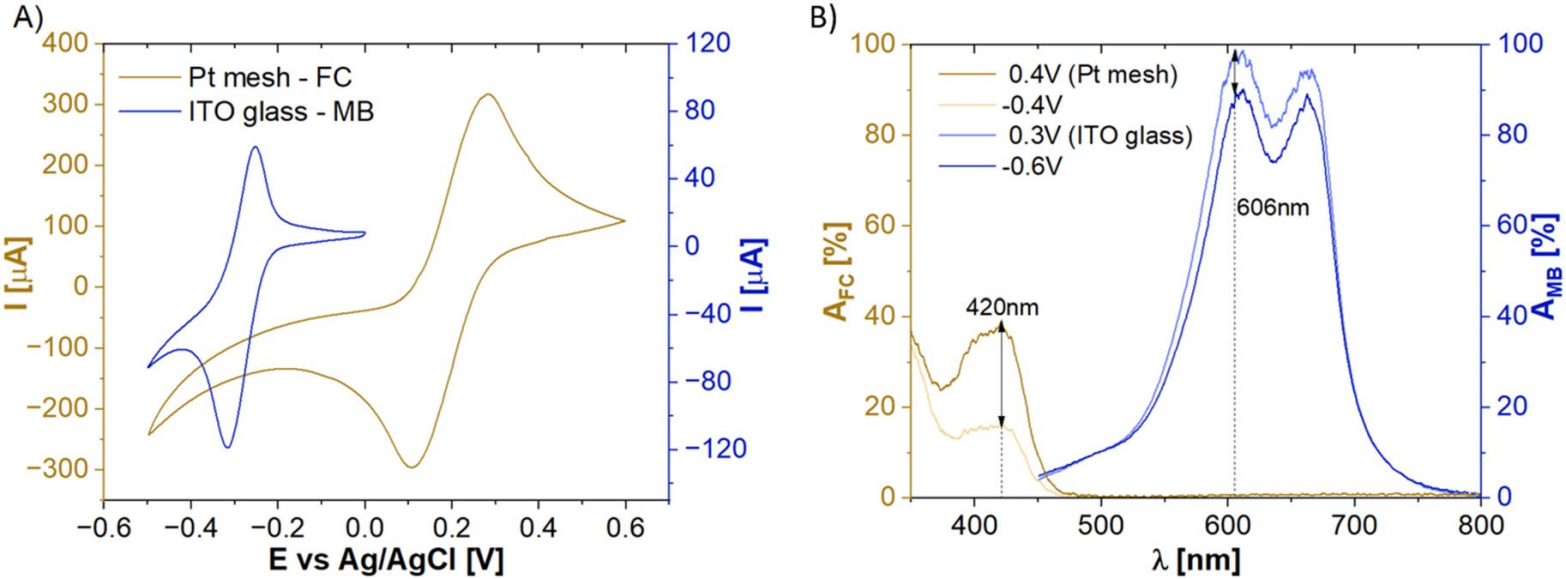
(**A**) Representative CV scans for Pt mesh in 1 mM FC and ITO glass in 0.125 mM MB identifying redox current peak position. (**B**) Absorbance spectra of the electrolytes containing FC and MB probes at potentials inducing their oxidation and reduction. Wavelengths at which maximum changes in the absorbance spectrum take place are marked for each of the solutions with black arrows.

First, the SEC system has been interrogated using two different standard WE, namely Pt mesh and ITO-coated glass slide. This part of the work aimed to identify suitable conditions for effective EC and optical measurements, such as, EC potential (*E*) range, redox probe concentrations, and spectral range of the interrogations. WEs were investigated individually with two redox probes, namely FC and MB as examples of anionic and cationic analytes, respectively. Both redox probes have well-defined, reversible EC responses that are accompanied by distinct changes in spectrum transmitted via the electrode/electrolyte. EC and optical properties of MB and FC were first investigated using CV as described in section “SEC and S2EC setups”. Figure 2A shows the exemplary results of CV measurements. FC (1 mM) was measured within the range of − 0.5 to + 0.6 V using Pt mesh. MB (0.125 mM) was investigated within the range of − 0.5 to 0.0 V using ITO-coated glass. The parameters have been chosen based on the analytes’ formal *E* ranges, electrode and material properties and utilized solvents^15^. All obtained CV curves show well-defined oxidation and reduction peaks observed at − 0.254 and − 0.316 V for MB and 0.282 and 0.106 V for FC, respectively. The peak-to-peak separation (*∆E*_*p*_) is equal to 62 and 176 mV for MB and FC, respectively. The anodic peak current to cathodic peak current ratio (*i*_*pa*_*/i*_*pc*_) in the case of FC is equal to 1, indicating *quassi*-reversible redox reaction. Conversely, for MB, the ratio smaller than 1 signifies an irreversible redox reaction. In general, the shape, position, and intensity of the oxidation/reduction peaks observed in CV scans are determined mostly by the electrode material, scan rate, concentration of electroactive species, diffusion coefficient, electrolyte composition and pH, adsorption, surface processes, as well as instrumental parameters. However, in this case, the differences which can be observed in Fig. 2A are mostly related to the type of electrode material, the electrode size, and the measured redox probe. Knowing the *E* corresponding to the redox current peaks for the chosen probes, the changes in optical spectrum (absorbance, *A*) at selected *E* could be further investigated. Figure 2B shows the exemplary results obtained for FC and MB when using Pt mesh and ITO-coated glass, respectively. By applying certain positive potentials (0.3 V and 0.4 V for ITO-coated glass and Pt mesh, respectively), the electrooxidation of the redox probes appears with maximum *A* changes at wavelength of 606 nm and 420 nm for MB and FC, respectively. When in turn a negative *E* is applied (− 0.6 V and − 0.4 V for ITO-coated glass and Pt mesh, respectively) both redox probes reduce to colorless forms, i.e., decrease in *A* is observed.

### Introduction of the LMR sensor towards S^2^EC

As proven in section “Identification of SEC measurement conditions”, thanks to high optical transparency, low electrical resistivity, and high EC activity ITO thin films can be used as an electrode material for SEC measurements. However, depending on the used ITO deposition method and its conditions, as well as applied substrate, the properties of the film may be tailored to obtain additional sensing effect, such as LMR^36,37^. LMR is a thin-film-based optical effect, which can take place at certain wavelength when relations between the electric permittivity of the film, substrate, and the external medium are fulfilled^38^. Tailored deposition conditions, including the process time have made a well-defined LMR minima in the output spectrum attainable^19,36,39^.

The ITO sensor was optimized to obtain the LMR and at the same time low resistivity of ITO required for EC. These were achieved for 35 mm of exposed core section and for 0.3 Pa and 90 min ITO deposition process parameters. It is worth mentioning that the properties of the LMR observed in the reflected spectrum strongly depend on both optical properties of the external medium and those of ITO. The later, depending on ITO properties, can be modulated in EC conditions as discussed in^19^. Therefore, the application of the LMR sensor as the WE within the SEC system can be considered as a source of additional information about the interactions taking place at the electrode surface and partially in its proximity (the cell volume). Having in mind, that the proposed sensor/electrode is based on a short section of a multimode fused silica optical fiber core coated with optimized ITO film, such “needle like” sensing solution can be considered as highly miniaturized comparing to the standard WE (ITO-glass or Pt mesh) what, in turn, reduces the volume of the analyzed sample and let’s adjust the sensor to any location within the volume of the analyte.

**Figure 3:**
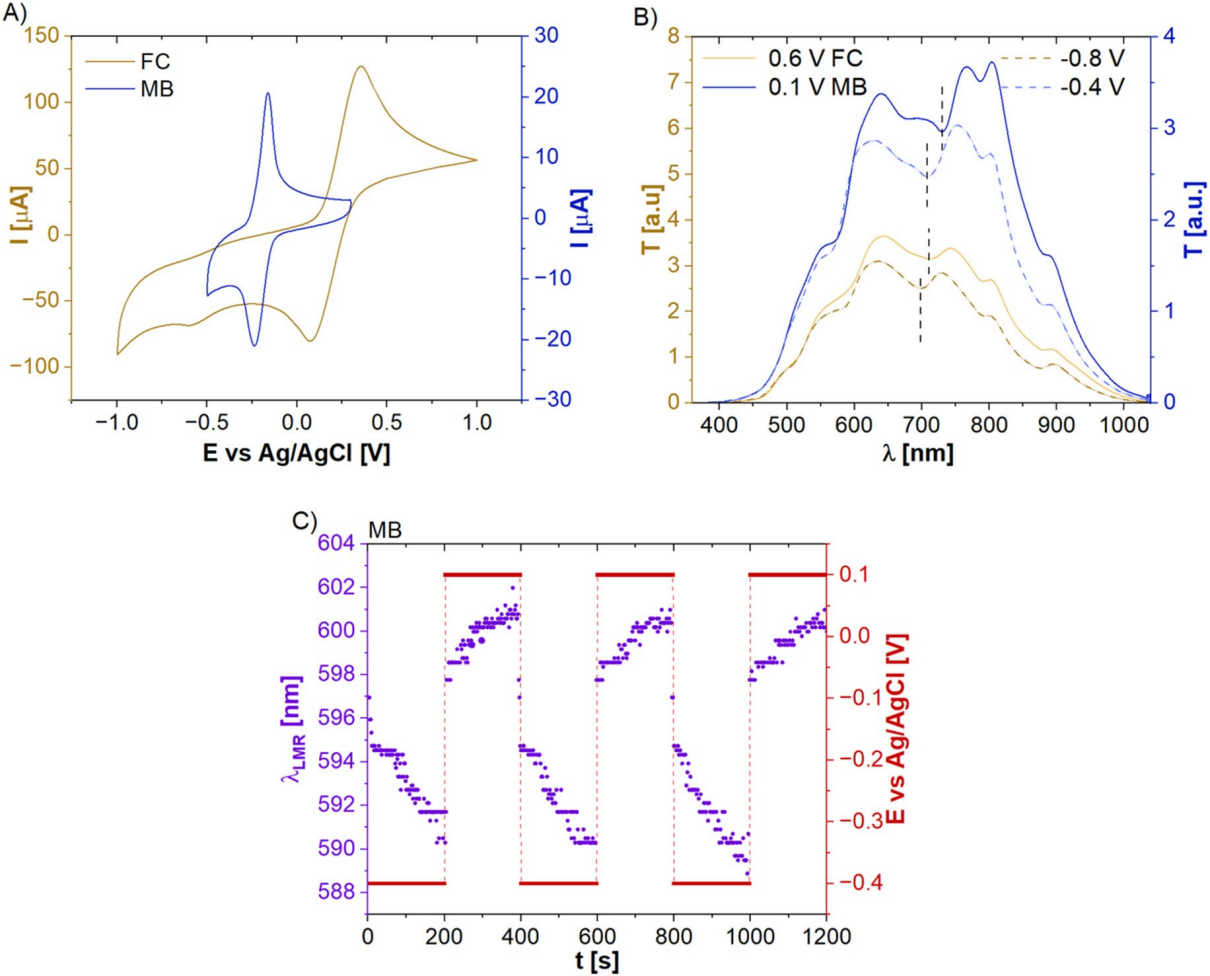
(**A**) CV scans for LMR sensor in MB and FC. (**B**) Evolution of LMR spectrum with *E* applied to induce oxidation and reduction of the redox probes. (**C**) A chronoamperometric response showing repeatable response of the LMR sensor in the MB solution.

At first, the LMR sensor had been EC characterized to verify its performance as the WE within the SEC setup. Figure 3A shows the results of CV measurements with both redox probes, namely FC and MB, and using the LMR sensors/electrode. As in the case of ITO-glass and Pt-mesh used in section “Identification of SEC measurement conditions”, obtained CVs show well-defined oxidation and reduction peaks at *E* reaching − 0.162 and − 0.236 V for MB and 0.356 and 0.074 V for FC, respectively. *∆E*_*p*_ is equal to 74 and 282 mV for MB and FC, respectively. In both cases, the *i*_*pa*_*/i*_*pc*_ ratio is higher than 1 indicating irreversible redox reactions. The obtained results clearly indicate that the ITO sensor effectively works as the WE in the EC setup. Despite the miniaturization of the electroactive area comparing to ITO-covered glass, still a relatively high current was measured (over 100 µA and over 20 µA in the case of FC and MB, respectively). It is also worth to mention that, except for the size differences between these two WE, also the substrate on which the ITO is deposited, its geometry, size, and material properties impacts the ITO film properties^36^. This reason justifies the observed difference in performance of the ITO-based WEs. Additionally, as for flat electrodes, optical response obtained with the ITO-LMR sensor was analyzed.

Figure 3B presents the differences in transmission spectra registered for both redox probes at different *E*. Based on the obtained oxidation and reduction peaks shown in Fig. 3A, FC has been interrogated by applying 0.6 V and − 0.8 V potential, while the MB has been interrogated by applying 0.1 V and − 0.4 V. Dash lines in Fig. 3B indicate the wavelength corresponding to the LMR. The changes in *E* are followed by λ_R_ and they are the combination of free charge density modulation within the ITO film as well as the RI changes of the solution surrounding the LMR sensor. Since ITO is a n-type semiconductor, by applying the negative potentials the accumulation of the electrons at the ITO-electrolyte interface occurs. Such change in electron density at the ITO surface is followed by the change of its optical properties as discussed in detail in^19^ and results in a shift of the spectrum towards shorter wavelengths. What is more, with the application of the negative *E* both redox probes reduce to colorless forms, what may further deepen the shift towards shorter wavelengths. Application of the positive *E* results in an opposite effect, decreasing the electron density at the ITO-electrolyte interface and oxidation of the probes. Thus, they restore to their color forms and shift the spectrum towards longer wavelengths. Moreover, as shown in Fig. 3C for the case of MB, λ_R_ shift caused by the applied *E* has some delay in following the *E* changes, but these are reversible. However, it must be noted that the LMR electrode properties remain stable during the EC processing. Moreover, it can be used to additionally monitor of both redox process and ITO surface properties. In case of any further modifications at the ITO surface such as its degradation or the formation of an additional film on it, modulation of the optical response would be significantly affected^19^.

Building upon this insight, the forthcoming cases will highlight a more pronounced distinction: the manifestation of optical changes requires more time to occur when compared to the nearly instantaneous electrical response. While electrical interactions reveal material reactions within a confined region near the electrodes or their surface, the optical response captures the gradual progression of the entire medium toward achieving thermodynamic equilibrium. The delay in the optical response is also attributed to the capacitance of ITO and its interface with the electrolyte. Here, charges accumulate over time and eventually stabilize^36^. Moreover, time-related discrepancies arise from both hardware limitations and software lags, leading to synchronization issues. The primary bottleneck in the setup is the amount of light reaching the detector, necessitating an extended interrogation time of 120 ms for channel 1 and 30 ms for channel 2. The speed of software-based data acquisition depends on the specific implementation and algorithm utilized. To enhance efficiency and reduce acquisition time, the development of the proposed system should encompass both hardware and software upgrades. This includes incorporating a higher-power light source, optimizing the setup to minimize optical losses, exploring alternative interrogation devices, enhancing the synchronization algorithm, and potentially offloading specific software-based tasks to the hardware level. However, it is important to note that these upgrades would lead to an overall increase in setup costs. Moreover, except for the above improvements, a more rapid optical response can be achieved with cost-efficient considerations involving optical losses. Firstly, reducing the volume of the analyte expedites the attainment of thermodynamic equilibrium. Secondly, employing more responsive materials enhances the speed of detecting changes in the refractive index. Additionally, combining a narrower optical beam with a low-volume container could enhance the efficiency of capturing optical changes and contribute to a swifter response.

### S^2^EC measurements

**Figure 4:**
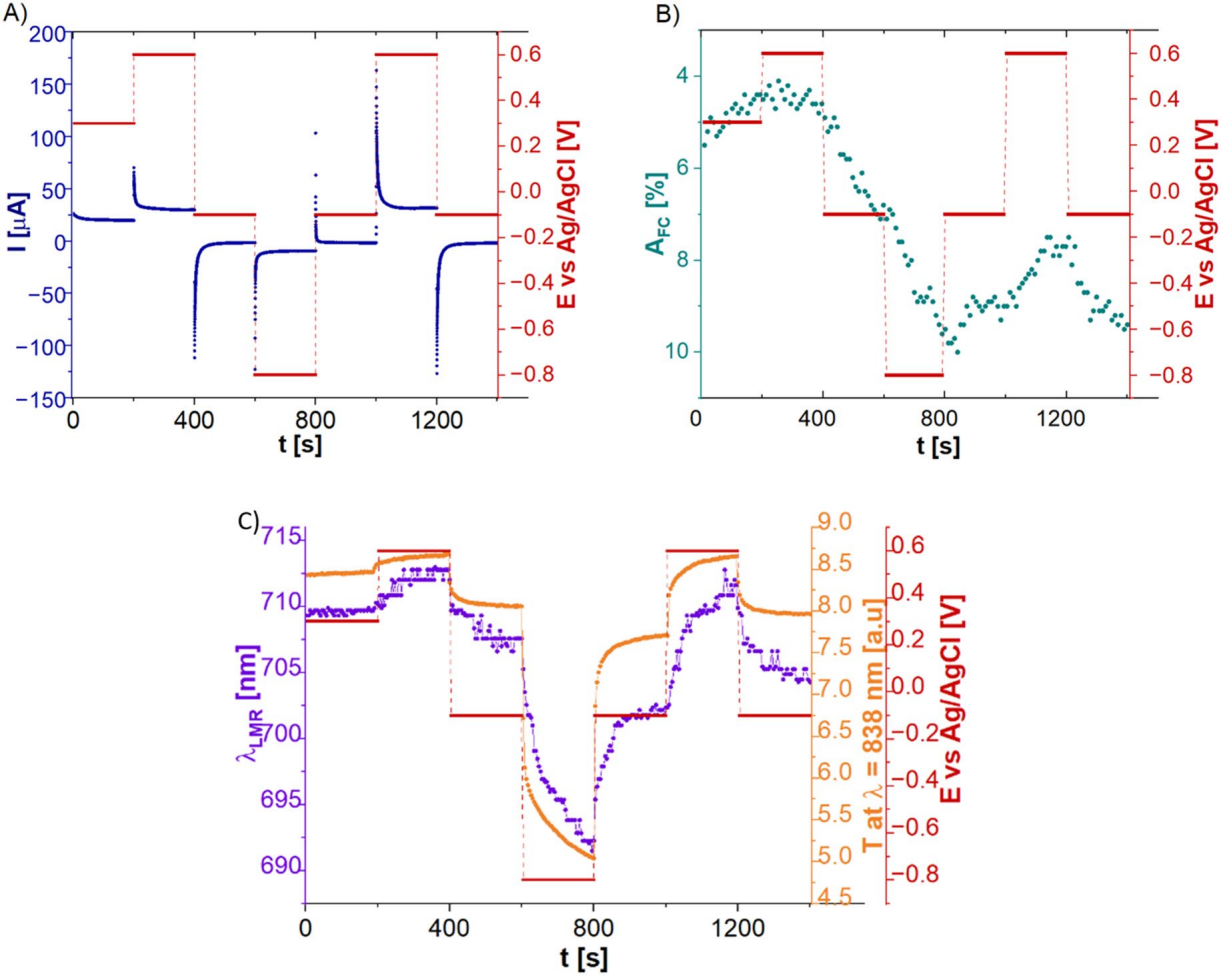
Results of the measurements in S^2^EC configuration for MSA, supported by LMR sensor, where (**A**) shows the EC current response, (**B**) absorbance at λ = 420 nm measured across the cell in Ch1, (**C**) LMR wavelength and transmission at 838 nm measured in Ch2.

Analysis reported in section “Introduction of the LMR sensor towards S2EC” facilitated application of the LMR sensor in the SEC setup as additional optical interrogator in Ch2 and formation of a S^2^EC system (Fig. 1). First, in the S^2^EC setup the FC solution has been analyzed. A set of data received as for standard SEC is shown in Fig. 4A and B.

Given the size of the measurement cell and miniaturized size of the WE, the CV measurements, due to their rapid character, were replaced with Multistep amperometry (MSA) where the certain potentials can be applied with the specified interval time. It allows the oxidized/reduced forms of the chosen redox probes to diffuse in the measurement cell’s volume, where they are measured in Ch1. Figure 4A depicts the obtained EC current response and Fig. 4B depicts *A* at λ = 420 nm measured in Ch1. It must be noted, that because the ITO-LMR sensor position vs the beam, Ch1 follows the changes of the analyte only. All the data obtained in EC and both optical channels show a clear influence of the *E* applied to the LMR sensor/electrode. While current follows well the *E* steps, some delay is again induced by the capacitance at the ITO-electrolyte interface. In the case of *A,* the changes appear only for specific *E*, i.e., for those changing the reaction type. Switch between oxidation and reduction or vice-versa affects the amount of reaction product absorbing the light in Ch1. For all other *E* in between the two—those that do not drive the redox reaction—the electrolyte remains ‘transparent’ and does not induce any noticeable changes in *A*. Moreover, *A* changes may not be fully reversible for the applied combination of the *E* steps. It is due to the limited velocity of induced redox reactions that is caused mainly by the diffusion to which, according to Lambert–Beer law, the *A* is proportional. The other reason is an unbalanced number of redox reactions that occurred due to the applied *E*. *A* irreversibility might have also stemmed from properties of the FC, in particular instability of its oxidized form^15^. Thanks to the application of the LMR sensor within the enhanced setup (S^2^EC), the response in Ch2 is additionally acquired. Figure 4C shows evolution of parameters corresponding to the LMR, namely λ_R_ and transmission (*T*) at a slope of the resonance (chosen at λ = 838 nm, T_838_). Both the T_838_ and λ_R_ change with the applied *E* and follow the pattern of *E*. First, the positive potential of 0.3 V was applied. As the potential value was very close to the formal redox potential (*E*^*0’*^*)* of the FC (0.25 V) the obtained response remained stable and was not changing in time. With the increase of the potential to 0.6 V slight shift of the λ_R_ to the longer wavelength was registered due to oxidation of FC to the Fe(CN)_6_^3−^ form which increases RI of the solution and causes color change to light yellow. Following decrease of the potential to − 0.1 V slightly shifted the λ_R_ towards shorter wavelengths what is expected to be mainly the influence of the free charge modulation inside the ITO layer. It partially also results from the FC reduction. However, due to the mentioned instability of the oxidized form it may not be treated as the dominant effect. The most significant changes were registered when the potential was stepped to more negative values, i.e., − 0.8 V. First, as it was reported in^40^, the more negative potential is applied to LMR, the larger are the charge distribution changes within the ITO layer. What is more, with such low potential applied, reduction back to the Fe(CN)_6_^4−^ (colorless form) may be expected. Therefore, over 10 nm shift towards shorter wavelengths corresponds to combined strong RI change within the ITO layer and solution’s RI decrease. While going back to the positive *E* (reduction to oxidation) opposite change of the spectra is seen, however, the trend is not fully reversible as the placement of the minimum differs from the one during the first cycle. Regarding the anionic character of the FC, the effect coming from electroimmobilization/electropolymerization at the LMR’s surface can be surely excluded. As discussed earlier in terms of *A*, the irreversibility of the process may arise from different reasons. However, the instability of the FC oxidized from, and therefore its higher diffusion rate^15^ resulting in different amount of reaction product may be the reason for less significant changes within the first cycle (oxidation to reduction).

**Figure 5:**
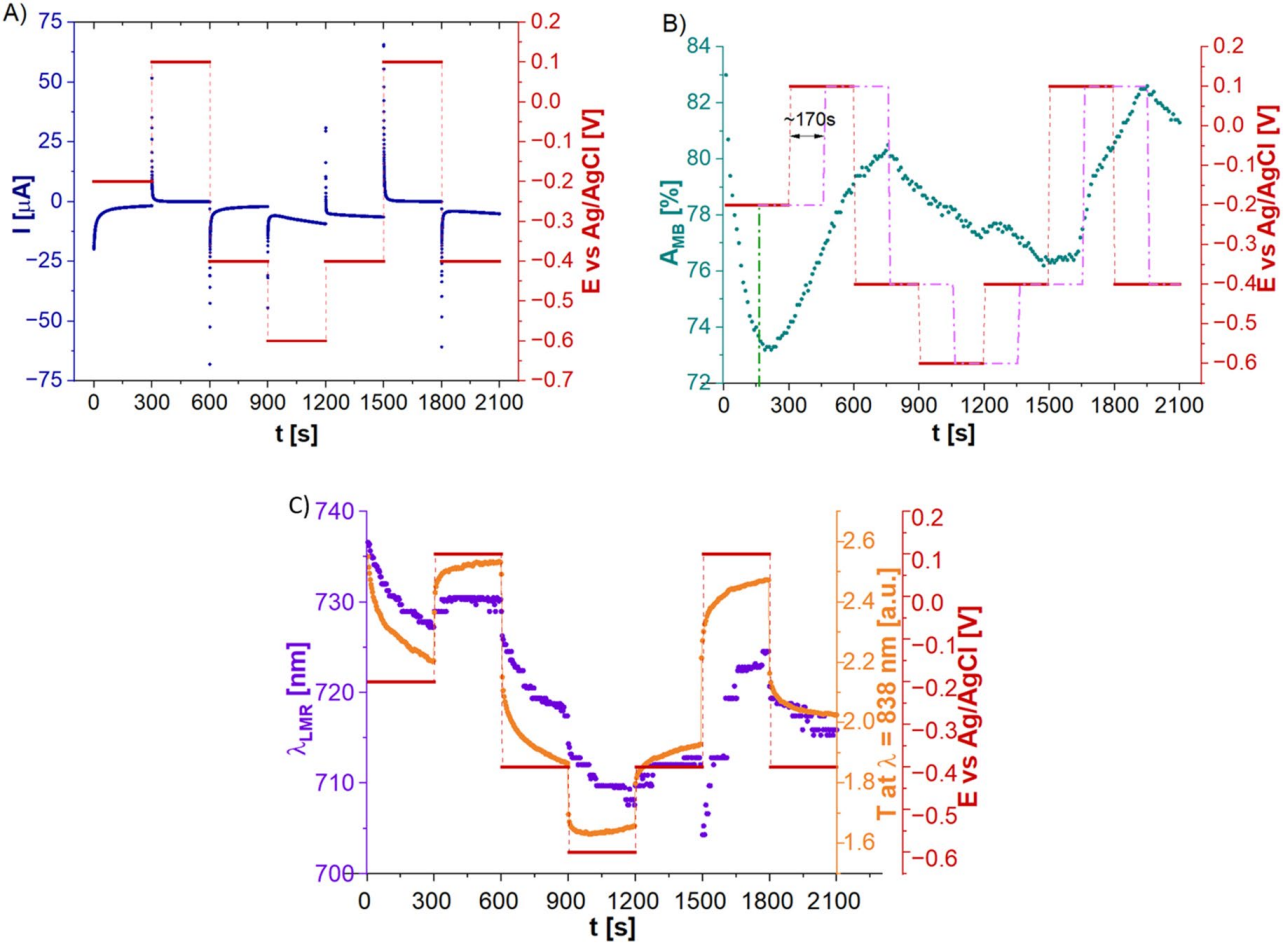
Results of the measurements in S^2^EC configuration for MSA, supported by the LMR sensor, where (**A**) shows results of EC current response, (**B**) A at λ = 606 nm measured across the cell, i.e., in Ch1, and (**C**) LMR wavelength and transmission at λ = 838 nm measured in Ch2.

The same setup has been also interrogated with the use of the MB as a redox probe. Figure 5 presents the obtained results. Same as in the case of FC, the impact of the *E* applied to the LMR sensor can be seen in EC response (Fig. 5A). However*,* the changes in MSA configuration can be spotted as the concentration as well as the properties of the MB significantly differs from FC. Considering smaller MB concentration, the applied time intervals were extended from 200 to 300 s, while the potential values had been adjusted to the redox reaction of MB. Again, for the same reasons as in the case of FC, some delays in Ch1 response, as well as the irreversible character of the *A* spectra can be noticed (Fig. 5B). Figure 5C shows the λ_R_ position and T_838_. First, the negative *E* of − 0.2 V was applied. The *E* value was very close to the E^0^′ of the MB, but over 10 nm shift towards shorter wavelength was clearly observed. As the MB remains at these conditions in its transparent form, it can be expected that the shift was induced mainly by the RI changes within the ITO layer. What is very interesting, applying the positive *E* = 0.1 V which drives the oxidation of the MB, and changes its transparent form to the blue one, should also change the properties of the redox probe, but there is no noticeable shift of the λ_R_ seen there. It may result from a small concentration of the MB, however, taking into account the anionic character of MB, it is possible that application of positive potential induced some electropolymerization of the oxidized MB form at the ITO-LMR’s surface. In this scenario, the RI change would not be recognized by the LMR sensor as its surface is partially blocked. Furthermore, the expected shift caused by the application of positive *E* could be compensated by the MB accumulation at the sensor’s surface. Further application of negative *E* shifts the λ_R_ towards shorter wavelengths. As in the case of FC, the combination of strong RI change within the ITO, along with solution’s RI change—as MB reduces in its transparent form—contribute to this effect. Start of a new MSA cycle and application of − 0.4 V induced very small changes in the λ_R,_ but it is justified by the small potential change, within which the MB does not change its form. Application of positive *E* (0.1 V) for the first approx. 100 s of the MSA cycle interestingly did not affect the LMR signal at all. Only after this time, the shift towards longer wavelengths could be observed indicating the RI change of the ITO layer, increased RI of the oxidized MB form, further electropolymerization of MB at the sensor’s surface or combination of all these effects.

As for FC, also in this case application of the ITO-LMR probe into the SEC setup as an additional optical interrogator (Ch2) and at the same time as a WE, turned out to provide numerous additional information about the redox probes and their behavior while applying different potentials. However, most importantly, in contrast to conventional SEC electrodes/setups, application of highly RI sensitive LMR probe except for following the RI of the solution, ease identification of even the smallest changes at the sensor’s/electrode’s surface. This can be advantageous when specific analyte’s immobilization needs to be detected. However, in this work, we focused on the characterization and exploration of new measurement possibilities offered by the ITO-LMR enhanced spectroelectrochemical measurement setup. Furthermore, we analyzed the basic mechanisms occurring in the opto-electrochemical setup and the interdependencies between the analyzed domains. Therefore, at this point, it is difficult to characterize the setup's sensitivity or resolution and, simultaneously, compare it with other sensing concepts.

## Conclusions

In this paper we presented an enhanced spectroelectrochemical measurement system with ITO-based lossy-mode resonance (LMR) optical fiber sensor. Due to optimized thickness and optical properties of the ITO film, the LMR was observed in the optical domain, while electrical properties of the ITO allowed for application of the sensor also as a working electrode in the electrochemical setup. The approach was demonstrated by examining two redox probes, namely potassium ferricyanide and methylene blue. As LMR strongly depends on both, the properties of the external medium and changes taking place at the sensor surface, the changes in applied potential were followed by both the shift of the resonance wavelength as well as transmission at specific wavelength. Moreover, the changes caused by the applied potential were highly reversible. “Needle like” form of the sensor is compact comparing to standard working electrodes, therefore provide a great flexibility in terms of placement of the sensor within the measurement system and allows to reduce the volume of the analyzed samples. What is more, fabrication of such sensor is scalable, highly reproducible, and low-cost. Enhancement of the spectroelectrochemical setup with the use of ITO-LMR increased the sensitivity of the analysis by adding supporting information about the state of the surface of the working electrode, the redox reaction itself, and by cross-verifying the obtained results. Such three-channel system can be applied in the future to other analysis but also to the sensing applications where the usage of the portable system is needed.

## Data Availability

The datasets used and analyzed during the current study available from the corresponding author on reasonable request.
